# Integrated effects of cold acclimation: physiological mechanisms, psychological adaptations, and potential applications

**DOI:** 10.3389/fphys.2025.1609348

**Published:** 2025-07-02

**Authors:** Yongxing Wang, Wei Liu, Dezhi Han, Yaqun Qiao, Weijing Sun, Conghui Wang, Xiaoyong Qin, Jinlong Xu

**Affiliations:** ^1^ Department of General Surgery, The 969th Hospital of the Chinese People’s Liberation Army Joint Logistics Support Force, Hohhot, China; ^2^ Department of Burn and Plastic Surgery, The 969th Hospital of the Chinese People’s Liberation Army Joint Logistics Support Force, Hohhot, China; ^3^ Clinical Laboratory, The 969th Hospital of the Chinese People’s Liberation Army Joint Logistics Support Force, Hohhot, China; ^4^ Department of Nuerology, The 969th Hospital of the Chinese People’s Liberation Army Joint Logistics Support Force, Hohhot, China

**Keywords:** athletic performance, cold acclimation, disease prevention, health promotion, physiological adaptation, psychological adaptation

## Abstract

Cold acclimation refers to the gradual process by which individuals adapt to cold environments. With the increasing impact of global climate change and the growing popularity of extreme sports, research on cold acclimation has attracted significant attention in fields such as sports medicine, psychology, and physiology. Evidence indicates that cold acclimation enhances athletic performance, promotes health, and contributes to disease prevention. However, much of the current research primarily examines the interplay between physiological mechanisms and psychological adaptations, with limited systematic evaluation of its broader effects. This review provides an in-depth analysis of the physiological adaptations associated with cold acclimation, including cardiovascular adjustments, metabolic regulation, and modification in immune responses. Additionally, the psychological effects of cold acclimation are examined, highlighting its potential to improve mental wellbeing. The review further explores practical applications of cold acclimation, such as optimizing athletic performance, promoting overall health, and mitigating disease risks. Finally, this paper identifies gaps in current knowledge and proposes future research directions to offer a theoretical framework and practical guidance for the expanded application of cold acclimation.

## 1 Introduction

Cold acclimation represents an adaptive response involving physiological and psychological changes in response to exposure to cold environments. The ongoing effects of global climate change and the increasing prevalence of extreme weather events have heightened the need to understand the impact of low temperature on human health. Cold acclimation impacts not only physiological adaptability but also psychological wellbeing. Previous studies have shown that in cold environments, short-term cold exposure (ranging from several minutes to a few weeks) leads to a significant increase in metabolic rate, driven by thermogenic demand (Cypess et al., 2013), and a marked elevation in heart rate, primarily mediated by sympathetic nervous system activation (Sramek et al., 2000). In contrast, long-term cold adaptation (lasting several months or more) generally results in a sustained elevation of metabolic rate, predominantly due to increased brown adipose tissue (BAT) activity. However, in some individuals, a slight decrease in metabolic rate may occur due to metabolic compensation. Resting heart rate may decrease over time, potentially reflecting enhanced parasympathetic tone or improved cardiac efficiency. These physiological responses should be interpreted in the context of individual variability and the intensity of cold exposure (Blondin et al., 2015). Psychologically, exposure to low temperatures has been associated with mood disturbances, including increased depression and anxiety, which, when combined with physiological discomfort, may adversely affect overall mental health.

The literature search was conducted using PubMed as the primary database. Keywords were derived from the core concepts of the research content, including “cold acclimation,” “cold adaptation,” “physiological mechanisms,” “psychological mechanisms,” “sports performance,” “health promotion,” and “disease prevention,” as well as their related combinations. The search time range was initially set from 2019 to 2024. However, due to the absence of some important studies published within the last 5 years, the search time range was expanded to from 2000 to 2024 to ensure the comprehensiveness and timeliness of the review.

### 1.1 Physiological mechanisms of cold acclimation

The physiological adaptations to cold acclimation are primarily mediated by the regulation of the neuroendocrine system and cellular responses. Exposure to low temperatures activates the body’s stress response, leading to the secretion of adrenocorticotropic hormone and other stress-related hormones, which enhance the ability to cope with cold-induced stress (Cypess et al., 2013). Additionally, cellular-level adaptations, including alterations in cell membrane fluidity and enzyme activity, have been observed in response to cold exposure. These changes are thought to improve cellular survival under low-temperature conditions (Blondin et al., 2015). Collectively, these mechanisms support the maintenance of physiological functions in cold environments, facilitating effective adaptation to external temperature variations.

### 1.2 Psychological adaptation to cold acclimation

The psychological effects of cold acclimation are significant, as exposure to cold environments have been associated with mood changes and alterations in social behavior. Cold exposure has been shown to enhance inflammatory responses, activating the NLRP3 inflammasome and elevating the levels of pro-inflammatory cytokines, such as IL-6 and TNF-α, that are positively correlated with the severity of depressive symptoms (Xia et al., 2023). Light therapy, involving daily exposure to bright light (10,000 lux) for 30 min, has been demonstrated to significantly alleviate symptoms of seasonal depression, with a reported efficacy rate of 68% (Terman and Su Terman, 2006). It has also been proposed that long-term cold-water swimming may reduce anxiety responses to cold stress by enhancing vagal tone. However, there remains ongoing debate and considerable individual variability. Cold adaptation protocols, such as regular mild cold exposure, may improve both metabolic function and mood by activating BAT, though excessive stress responses should be avoided (Søberg et al., 2021). These psychological changes can negatively impact not only social engagement but also work performance and cognitive efficiency. Therefore, understanding the psychological effects of low temperatures is essential for devising effective coping strategies. Interventions focused on psychological support are particularly critical in enabling individuals to adapt more effectively to the challenges posed by cold environments.

### 1.3 Applications of cold acclimation across fields

The study of cold acclimation has garnered significant interest across various disciplines due to its wide-ranging practical applications. In the medical domain, cold acclimation has been investigated as a basis for innovative treatments, such as cryotherapy, which is utilized to reduce inflammation and alleviate pain (Sharma et al., 2023). In sports medicine, cold acclimation techniques are employed to enhance athletes’ endurance and recovery, enabling enhanced physical and psychological performance during competitive events. Furthermore, research on cold acclimation has contributed to agricultural and ecological research, providing insights into how plants and animals adapt, survive, and reproduce under extreme environmental conditions (Vera Hernández et al., 2023).

In conclusion, the physiological and psychological impacts of cold acclimation are extensive, involving complex mechanisms and offering valuable applications in diverse fields. Continued research into cold acclimation is expected to yield more effective strategies and interventions, aiding individuals in adapting to dynamic environmental conditions.

## 2 Physiological mechanisms of cold acclimation

### 2.1 Thermoregulation and heat balance

The physiological effects of cold environments are predominantly observed in thermoregulation and heat balance mechanisms. Thermoregulation is a crucial physiological process that maintains internal environmental stability, involving complex interactions between the nervous, endocrine, and circulatory systems. In response to cold exposure, the hypothalamic temperature regulation center is activated, initiating heat-generating processes such as shivering and peripheral vasoconstriction to minimize heat loss (Haman et al., 2022).

Individuals who undergo cold acclimation demonstrate enhanced heat production capabilities and improved heat retention, thereby maintaining core body temperature stability in cold environments. These adaptive changes are not confined to the regulation of physiological functions but may also include modifications in gene expression that contribute to increased. Such mechanisms underscore the body’s ability to adapt to cold environments through coordinated physiological and molecular processes (Domínguez-Guerrero et al., 2019).

### 2.2 Vascular response and circulatory adaptation

Vascular responses and circulatory adaptations play a critical role in maintaining body temperature in cold environments. Cold exposure triggers peripheral vasoconstriction, which reduces blood flow to the skin and extremities, thereby preserving core body temperature. This immediate physiological adaptation is complemented by long-term acclimation processes. Research indicates that chronic exposure to cold conditions induces structural and functional adaptations in the vascular system, including increased vascular wall thickness and improved endothelial function, which enable more effective regulation of blood flow and blood pressure (Tveita and Sieck, 2022). Additionally, circulatory adaptations to cold environments also involve modifications in heart rate and cardiac output, ensuring the delivery of oxygen and nutrients necessary for physiological functions in low-temperature conditions (Bychkov et al., 2020).

### 2.3 Metabolic adaptations

Cold acclimation is also associated with significant metabolic adaptations to meet the increased energy demands required to maintain body temperature in low-temperature environments. The basal metabolic rate of organisms typically increases to generate more heat, supporting thermoregulation under low-temperature conditions. This process involves the regulation of various biochemical pathways, including the oxidation of fatty acids and the breakdown of glycogen (Lamptey et al., 2021). Studies have found that individuals exposed to cold environments for extended periods exhibit improved mitochondrial function and greater energy metabolism efficiency (King et al., 2022). Furthermore, low temperatures affect hormone levels, such as the secretion of adrenaline and thyroid hormones, further enhancing metabolic activity. These physiological and metabolic adjustments not only facilitate survival in cold environments but also provide insights into the physiological effects of low temperatures (Ohnishi et al., 2024).

## 3 Effects of cold acclimation on the cardiovascular system

### 3.1 Heart rate variability (HRV) and blood pressure regulation

Cold acclimation significantly impacts the cardiovascular system, particularly through its effects on HRV and blood pressure regulation. Evidence suggests that exposure to cold environments induces adaptive changes in the autonomic nervous system, influencing the regulation of heart rate and blood pressure. HRV serves as a key indicator of autonomic nervous system regulation of the heart, with higher HRV typically reflecting improved cardiovascular health. During the initial stages of cold acclimation, sympathetic nervous activity is enhanced, which may lead to a reduction in HRV, a natural short-term response to cold stimuli. However, with prolonged adaptation, HRV may stabilize or even improve, indicating an enhanced adaptability and health status of the cardiovascular system (Shafiq et al., 2023).

Cold acclimation also impacts blood pressure variability. Research has demonstrated that exposure to low temperatures can increase the high-frequency variability of blood pressure, which is related to heightened sympathetic nervous system activity (van Wingerden et al., 2024). These changes highlight that cold acclimation involves not only physiological adaptations to the cold but also significant adjustments in cardiovascular function.

### 3.2 Changes in hemorheological characteristics

Cold acclimation significantly affects hemorheological characteristics. These characteristics such as blood viscosity, red blood cell aggregation, and deformability, directly influence blood flow and cardiovascular health. Studies indicate that initial exposure to cold environments may lead to increased blood viscosity, resulting in decreased blood flow and heightened cardiac workload (Teległów et al., 2024). Furthermore, cold acclimation may enhance red blood cell aggregation, further impacting the efficiency of blood circulation.

While these changes may pose short-term challenges, long-term cold acclimation is associated with gradual improvements in hemorheological characteristics. Enhanced red blood cell deformability and increased blood flow are often observed, potentially contributing to better cardiovascular function over time (Valeanu et al., 2021). Long-term winter swimmers (n = 50) exhibit an 8% reduction in blood viscosity and decreased fibrinogen levels, which are associated with reduced red blood cell aggregation (Srámek et al., 2000). Therefore, understanding the impact of cold exposure on hemorheological characteristics is crucial for evaluating its implications for cardiovascular health and the potential therapeutic benefits of cold exposure.

### 3.3 Cardiovascular health benefits of long-term adaptation

Acute cold exposure can increase the risk of myocardial infarction and stroke (Gasparrini et al., 2017). Previous evidence has even indicated that cold-related cardiovascular deaths account for 40%–55% of excess winter mortality in temperate regions worldwide (Analitis et al., 2008). Prolonged cold acclimation has been associated with significant cardiovascular health benefits. Chronic cold exposure can activate BAT, promoting lipid oxidation and improving insulin sensitivity (GBD, 2013 Mortality and Causes of Death Collaborators, 2015). It also enhances vascular endothelial function (GBD, 2019 Risk Factors Collaborators, 2020), and may lower resting heart rate and blood pressure, thereby contributing to improved cardiovascular function (Global Burden of Disease Study, 2013 Collabora tors, 2015). Research indicates that adapting to cold environments enhances cardiac pumping efficiency and vascular elasticity, thereby reducing the risk of cardiovascular diseases (Ashcroft et al., 2024). Additionally, cold acclimation has been shown to improve heart rate variability, strengthening the regulatory capacity of the autonomic nervous system, which contributes to overall cardiovascular health. Individuals with long-term exposure to cold temperatures exhibit increased cardiovascular resilience and adaptability in response to environmental changes. These physiological adaptations not only support improved athletic performance but also reduce the risk of cardiovascular events in daily life (Tonhajzerova et al., 2021). Consequently, cold acclimation serves not only an environmental adaptation but also as a valuable strategy for promoting cardiovascular health.

## 4 Cold acclimation and immune function

### 4.1 Changes in immune cell activity

Cold acclimation significantly influences immune cell activity, modulating immune responses through neural signaling pathways in both short-term and long-term cold exposure. For instance, studies in rodent models have demonstrated that exposure to low temperatures induces a stress response, characterized by the release of cytokines and subsequent activation of thermogenesis-associated BAT. This process involves changes in the activity of γδ T cells, suggesting a regulatory role during the adaptation to cold environments (Vasek et al., 2024).

Additionally, research on the effects of regular exposure to cold, such as cold showers, have shown significantly higher levels of immunoglobulins among participants, indicating enhanced humoral and cell-mediated immune responses (El-Ansary et al., 2024). hese findings suggest that cold acclimation impacts not only the quantity of immune cells but also their functional state, thereby strengthening the body’s resistance against pathogens.

### 4.2 Regulation of inflammatory responses

Cold acclimation plays a significant role in regulating inflammatory responses. Research indicates that exposure to low temperatures suppresses the release of specific inflammatory mediators, thereby reducing inflammation. For instance, cold exposure has been associated with reduced expression of major histocompatibility complex class II on monocytes, which diminishes the initial activation and pathogenic potential of T cells. This phenomenon may have potential clinical significance in the prevention and treatment of autoimmune diseases (Spiljar et al., 2021).

Additionally, cold exposure has been shown to enhance the activity of antioxidant enzymes, reducing oxidative damage associated with inflammation. This mechanism provides protection against the effects of chronic inflammation and may also benefit the body during acute inflammatory responses (Zhang et al., 2022). By decreasing the prevalence of chronic inflammation and offering protective effects during acute episodes, cold acclimation emerges as a potential therapeutic approach for managing inflammatory conditions.

### 4.3 Impact on infection risk

The impact of cold acclimation on infection risk is multifaceted. Moderate cold exposure can enhance immune function through the adrenaline-IL-6 axis, which boosts phagocyte activity via the STAT3 pathway (GBD, 2019 Diseases and Injuries Collaborators, 2020) and through metabolic reprogramming. Cold-induced fatty acid oxidation supports the survival of memory T cells, thereby helping to reduce infection rates (GBD, 2019 Risk Factors Collaborators, 2020). For instance, regular exposure to cold environments, like cold showers, has been associated with higher levels of immunoglobulins, which may enhance resistance to infections (El-Ansary et al., 2024). However, excessive or prolonged exposure to cold conditions may suppress immune function. This immunosuppression is thought to occur through activation of the cortisol-myeloid-derived suppressor cell axis and depletion of energy resources, ultimately increasing susceptibility to infections. In certain animal models, changes in the expression of immune-related genes following cold exposure have been observed, suggesting that during colder seasons, immune system regulation may heighten vulnerability to infections (Vaziri et al., 2024). Therefore, moderate cold acclimation may reduce infection risk by enhancing immune responses. While moderate acclimation can bolster immune responses and reduce infection likelihood, careful regulation of cold exposure intensity and duration is essential to prevent adverse effects on immune function. We also acknowledge potential limitations of our study, particularly regarding human data. Although our findings offer valuable insights, their translation to human physiological responses remains uncertain. We have made efforts to distinguish results derived from animal models from their potential implications for human immunity, underscoring the need for further research in human populations to validate and expand upon our findings.

## 5 Cold acclimation and psychological adaptation

### 5.1 Psychological resilience and adaptability

Psychological resilience is defined as the ability to adapt to and recover from stress, adversity, or challenges. Evidence suggests that psychological resilience is closely related to an individual’s adaptability, especially in cold environments, where resilience plays a critical role. During cold acclimation, individuals encounter both physical and psychological challenges, and resilience enables them to manage these challenges more effectively. For instance, individuals with higher psychological resilience demonstrate a faster adjustment to environmental changes and are more likely to maintain emotional stability during exposure to cold, thereby reducing anxiety and depression symptoms that may arise due to low temperatures.

This adaptive capacity not only supports mental health but also contributes to physiological adaptation by promoting the body’s tolerance to cold (Scharte, 2024). Additionally, psychological resilience interacts with factors such as social support and emotional regulation, collectively shaping an individual’s ability to adapt successfully to cold conditions.

### 5.2 Impact of cold exposure on emotions

The impact of cold exposure on emotions is a multifaceted process. Studies indicate that exposure to cold environments can lead to significant emotional fluctuations. Prolonged exposure, especially in the absence of adequate coping mechanisms, may induce negative emotions such as sadness, anxiety, and depression. Cold environments affect emotional wellbeing both directly, through physical discomfort, and indirectly, by influencing psychological states. For instance, physical discomfort caused by cold exposure can lead to emotional unrest and increased irritability (Xu et al., 2023). However, moderate exposure to cold has been observed to foster emotional adaptation, enhancing psychological resilience and enabling individuals to maintain a positive emotional state when encountering adverse conditions. Therefore, managing the negative emotional effects of cold exposure and fostering resilience are critical components of successful psychological adaptation to cold environments.

### 5.3 Social support and mental health

Social support is a vital determinant of mental health, particularly during the process of cold acclimation, where it significantly impacts an individual’s adaptability. Research has shown that a strong social support network alleviates the psychological stress caused by cold exposure and enhances their psychological resilience, facilitating better coping mechanisms in cold environments (Rakap and Vural-Batik, 2024). For instance, emotional and practical support from family, friends, or colleagues provides individuals with a sense of comfort and reduces feelings of isolation during exposure to cold temperatures. Additionally, social support promotes positive emotions and increases one’s confidence in overcoming adversity. Establishing and maintaining a strong social support system is therefore essential for improving psychological adaptability and promoting mental health during cold acclimation.

## 6 Cold acclimation applications and prospects

### 6.1 Application in sports performance

The application of cold acclimation to enhance sports performance has garnered increasing interest, particularly in endurance and high-intensity training. Research has demonstrated that moderate cold exposure induces a range of physiological adaptations that can enhance athletic performance. For instance, cold exposure stimulates mitochondrial biogenesis within muscle tissue, resulting in enhanced energy metabolism efficiency and improved overall performance.

Additionally, training in cold environments can improve athletes’ endurance and recovery capabilities, reducing muscle damage and fatigue associated with strenuous exercise (Liu et al., 2022). Cold acclimation has also been observed to optimize cardiovascular responses, including heart rate and blood pressure regulation, which are critical for high-intensity exercise performance (Wu et al., 2021). Despite these advancements, further research is necessary to elucidate the underlying mechanisms of cold acclimation and to develop evidence-based, personalized training regimens for athletes.

### 6.2 Potential in health promotion and disease prevention

Cold acclimation has shown promise not only in enhancing sports performance but also in promoting health and preventing disease. Research has found that cold exposure activates BAT, leading to increased energy expenditure, which supports weight management and improves metabolic health. The method involves exposing the body to a low-temperature environment of 10 to 15°C for 1–2 h per session, conducted 3 to 5 times per week (GBD, 2013 Mortality and Causes of Death Collaborators, 2015; GBD, 2019 Risk Factors Collaborators, 2020; van d et al., 2014; Braadland et al., 2019). However, it is important to note that these conditions are based on current studies and may vary depending on individual differences (Gasparrini et al., 2017; Analitis et al., 2008). Several clinical trials have demonstrated that cold acclimation can provide lasting metabolic benefits for the human body. For example, previous research has shown improvements in insulin sensitivity, glucose metabolism, and lipid profiles (GBD, 2019 Risk Factors Collaborators, 2020; van d et al., 2014; Cypess et al., 2015; Yoneshiro et al., 2013). Additionally, cold environments have beneficial effects on the immune system, enhancing resistance to infections and potentially reducing the risk of chronic diseases (Sos et al., 2021). In terms of mental health, cold acclimation may improve mood, reduce symptoms of anxiety and depression, and promote overall psychological wellbeing (Rocha et al., 2022). However, the application of cold acclimation in health promotion and disease prevention requires careful consideration of individual variability and adaptability to ensure both safety and efficacy.

### 6.3 Future research directions and challenges

Research on cold acclimation presents a range of challenges and opportunities for future exploration. A key priority is to elucidate the underlying physiological mechanisms, including the role of gene expression and metabolic pathways in the body’s response to low temperatures (Li et al., 2023). Additionally, understanding individual differences in cold acclimation is essential for developing tailored adaptation strategies for specific populations, such as athletes, older adults, and those with chronic conditions (Tsai et al., 2020). Systematic evaluation of the long-term effects and potential risks of cold acclimation is also necessary to ensure its safe application in health promotion and disease prevention (Xu et al., 2020).

Furthermore, the escalating impact of climate change underscores the importance of studying cold acclimation as a means of safeguarding human health in extreme climate conditions. Research should focus on devising effective strategies to adapt to environmental changes, providing a foundation for mitigating the health risks associated with fluctuating temperatures (Ebi et al., 2023).

## 7 Conclusion

Cold acclimation plays a crucial role in facilitating both physiological and psychological adaptation to cold environments. Evidence from existing studies indicates that exposure to cold stimuli not only enhances athletic performance but also promotes health and aids in disease prevention. Research in this field has advanced considerably, elucidating mechanisms such as metabolic regulation, immune system activation, and improvements in psychological wellbeing. However, despite the growing body of research supporting the benefits of cold acclimation, discrepancies persist, highlighting the need for further investigation.

One critical area of focus is the influence of individual differences on the efficacy of cold acclimation. Variations in physiological characteristics, psychological states, and adaptability among individuals may result in different outcomes following exposure to cold environments. To address these variations, future research should prioritize individualized approaches to better elucidate the underlying mechanisms and optimize the application of cold acclimation for diverse populations.

Moreover, the potential applications of cold acclimation across diverse populations warrant further investigation. While existing research predominantly focuses on athletes and healthy individuals, limited attention has been given to specific groups, such as older adults and individuals with chronic health conditions. More clinical studies are needed to investigate the underlying adaptation mechanisms, evaluate the effects, consider the importance of individual factors such as age, sex, and baseline health status, and identify potential risks of cold acclimation in these populations. Such efforts are essential to ensure its safe and effective implementation.

In summary, cold adaptation is a promising area of research. Although current data have yet to directly reveal the neurohormonal pathways linking physiological and psychological domains, we speculate that a potential connection exists between the adrenal medulla and the brain. This connection may influence the overall effects of cold adaptation, carrying significant implications for both physiological and psychological adjustment. To maximize its potential for widespread application, future research should address individual variability and the specific needs of diverse populations. Advancing this field will require integrating different research perspectives and findings to establish a unified framework, thereby providing a robust foundation for both the theoretical and practical applications of cold acclimation. Cold adaptation is a double-edged sword, requiring careful application under medical supervision, individualized protocols, and risk stratification. Future research should focus on identifying early warning signs in high-risk populations and optimizing intervention strategies.
